# Investigating the Mechanisms of Discoloration in Modern Dental Materials: A Comprehensive Characterization Approach

**DOI:** 10.3390/jfb15090246

**Published:** 2024-08-27

**Authors:** Maria Gawriołek, Naisargi Varma, Amadeusz Hernik, Wojciech Eliasz, Marta Strykowska, Elżbieta Paszyńska, Beata Czarnecka, Marek Sikorski

**Affiliations:** 1 Department of Integrated Dentistry, Poznan University of Medical Sciences, 61-701 Poznan, Poland; m.gawriolek@ump.edu.pl (M.G.); ahernik@ump.edu.pl (A.H.); paszynska@ump.edu.pl (E.P.); 2 Faculty of Chemistry, Adam Mickiewicz University, 61-614 Poznan, Poland; varmanishu238@gmail.com (N.V.); sikorski@amu.edu.pl (M.S.); 3 Department of Conservative Dentistry and Endodontics, Poznan University of Medical Sciences, 61-701 Poznan, Poland; 4 Department of Biomaterials and Experimental Dentistry, Poznan University of Medical Sciences, 61-701 Poznan, Poland; martastrykowska@ump.edu.pl (M.S.); czarnecka@ump.edu.pl (B.C.)

**Keywords:** dental composite, color parameters, optical microscopy, surface defects

## Abstract

In general, patients’ opinions on reaching ideal esthetics while restoring dental tissues is one of the most important part of the oral treatment. Unfortunately, discoloration of dental materials may occur due to intrinsic and extrinsic factors. The aim of the study was to evaluate the color stability of frequently used dental resin materials and determine the mechanism of their discoloration. The study used various characterization techniques (optical microscopy, Fourier-transform infrared spectroscopy, low-temperature N_2_ adsorption, diffuse reflectance spectroscopy, and luminescence) to understand the effect of surface defects on discoloration. The adsorption of model liquids on the surface was confirmed to be related to the increase in BET surface area. The study found that the adsorption of discolorants, such as coffee, tea, and wine, on the surface of the dental material follows the multilayer BET model. When the surface is smooth, the discoloration is usually within acceptable limits, with a maximum of ∆E = 3.3. The discoloration made by tea and demineralized water was within acceptable limits even after 7 days of exposure.

## 1. Introduction

Smiling plays a crucial role in human interactions, and aesthetic dentistry aims to create an artificial dentition that closely resembles natural teeth. Color is an important aspect of dental restorations, which should coordinate with the patient’s gingivae, lips, and face. Reaching good esthetics while restoring dental tissues is often the most important part of the treatment for the patient. Discoloration of dental materials can occur due to intrinsic and extrinsic factors. The objective study of color in dentistry began in the 1980s [1] and led to the development of colorimetry standards. This was mainly achieved by international organizations such as CIE (Commission Internationale de l’Eclairage) in Europe and NBS (National Bureau of Standards) in the USA, which have introduced a number of norms and modified and unified the CIE Lab system. The CIE Lab system, which uses L*, a*, and b* coordinates, describes color in dentistry. Color stability is a general problem for dental materials; the evaluation of discoloration of dental materials is still important issue [2,3,4,5,6]. Some authors focus on the maximum acceptable color difference in dentistry, with values ranging from ΔE = 1.5 [7] and ΔE = 2 [8,9] to ΔE = 3.3 [9,10,11,12]. Liberal approaches propose values up to ΔE = 3.7 [13,14,15,16]. Studies have explored the color stability of prosthetic materials, including color matching with dental color keys and the impact of discoloring elements such as tea, coffee, and red wine [5,6,7,8,9]. Red wine was found to cause the largest color difference, while coffee and tea caused a lower degree of discoloration. Additionally, the hydration of dental materials resulted in statistically significant color changes, and coffee and tea produced more discoloration than distilled water. Red wine produced a unique discoloration effect, characterized by an increased contribution of blue and green [10,11,12].

The aim of the investigation was to examine the color stability of five modern dental materials, G-ænial A’chord, G-ænial Universal Injectable, G-ænial Anterior, Icon, Filtek™ Ultimate, and to determine the mechanism of their discoloration.

## 2. Materials and Methods

### 2.1. Materials

Five dental materials were chosen for this study. Each of the selected materials has specific properties that make it most suited for a variety of indications in restorative dentistry. The composite materials included G-ænial A’chord, G-ænial Universal Injectable, G-ænial Anterior, Icon, and Filtek™ Ultimate. The disc-shaped samples were made of each of the materials studied using a specially prepared transparent plastic mold. All samples were produced in licensed dental laboratories strictly following the procedural and technological requirements. The diameter of the disc samples was 5 mm and 1.5 mm thick. The samples were polished with Sof-Lex XT (extra thin) finishing and polishing discs (3 M) in the correct sequence (coarse, medium, fine, and superfine) according to polishing protocol. A total of 10 samples of each molded composite were analyzed to eliminate manufacturing errors.

G-ænial A’chord (GC Corporation, Tokyo, Japan) is a versatile composite restorative that simplifies the process of achieving aesthetically pleasing dental restorations. With its unishade system consisting of only five core shades, it can replicate the 16 classic Vita shades. The material also creates natural fluorescence and mimics the reflection of natural light, resulting in virtually invisible restorations even in special lighting conditions. G-ænial A’chord is a highly esthetic dental composite material specifically designed for posterior restorations. G-ænial A’chord incorporates a proprietary filler technology that provides enhanced strength, wear resistance, and polishability. Gænial A’chord is primarily used for posterior restorations, including class I and class II cavities. Its high strength, wear resistance, and excellent polishability make it suitable for withstanding the occlusal forces and ensuring long-lasting restorations in the posterior region.

G-ænial Universal Injectable (GC Corporation, Tokyo, Japan) is a high-strength universal restorative composite designed for long-lasting and aesthetically pleasing dental restorations. Its strength and resistance come from its high load of ultrafine barium particles and GC’s full-coverage silane coating (FSC) technology. G-ænial Universal Injectable is suitable for all cavity classes without the need for a covering layer. Furthermore, it has a high radiopacity of 252%, making it easy to follow up on restorations and detect any secondary caries. Gænial Universal Injectable is particularly useful for minimally invasive restorations. The material offers excellent adaptability, allowing for precise adaptation to cavity walls and complex anatomical contours. G-ænial Universal Injectable demonstrates optimal translucency and color range, ensuring seamless integration with the surrounding dentition. Its flowable consistency allows for easy adaptation to cavity walls and complex anatomical contours, making it suitable for small- to moderate-sized restorations, including class III and class V cavities. The injectable nature of G-ænial Universal Injectable makes it convenient for use in pediatric dentistry, where handling and placement can be challenging. It can be utilized for minimally invasive restorations and repairing small enamel defects in children.

G-ænial Anterior (GC Corporation, Tokyo, Japan) is a universal composite restorative material with a simplified unishade system for anterior and posterior restorations. G-ænial Anterior offers natural fluorescence created by its unique filler technology that mimics the reflection of natural light which results in invisible restorations both in natural daylight and in special lighting conditions (e.g., discotheques). The combination of a Bis-MEPP monomer (bisphenol-A ethoxylate dimethacrylate) and optimized filler–monomer allows the dentist to easily sculpt the material with a hand instrument or a brush, and full-coverage silane coating (FSC) and High Performance Pulverized CERASMART (HPC) gives the material its high strength, low wear, gloss retention, low discoloration and excellent radiopacity. G-ænial Anterior is a light-cured, nanohybrid dental composite material primarily used for anterior restorations, where excellent esthetics and shade matching are critical. It is indicated for class III, class IV, and class V cavities in the anterior region, enabling the creation of natural-looking restorations that blend in with the surrounding dentition.

Icon Icon (DMG, Hamburg, Germany) is a material to treat incipient caries: it is an infiltrant of caries. This micro-invasive material fills and reinforces demineralized enamel without drilling or anesthesia, up to the first third of dentin (D1). Icon is primarily indicated for the treatment of white spot lesions, such as those resulting from incipient caries or demineralization. It infiltrates and reinforces the porous enamel, arresting the carious process and improving the appearance of the affected tooth. Icon provides a minimally invasive alternative to traditional restorative materials for treating early carious lesions.

Filtek™ Ultimate (3M ESPE, St. Paul, MN, USA) is a visible light-activated composite designed for use in anterior and posterior restorations. A dental adhesive, such as those manufactured by 3 M ESPE, is used to permanently bond the restoration to the tooth structure. Filtek™ Ultimate is widely used for esthetic restorations due to its excellent esthetic properties and color-matching capabilities. It is indicated for anterior restorations (class III, class IV) as well as posterior restorations (class I, class II) where esthetics are a priority.

The samples were light-cured using a 1000 mW/cm^2^ curing unit (LED-E, Woodpecker, Guilin, China), and the curing times were as follows: G-ænial A’chord was cured for 10 s, G-ænial Universal Injectable for 10 s, G-ænial Anterior for 20 s, Icon for 40 s, and Filtek™ Ultimate for 20 s.

### 2.2. Test Liquids

Tests on the dental materials were performed using the following model liquids.

Demineralized water: Demineralized water was used for control.

Red wine: Red wine (semi-sweet) was used as commercially available: Kadarka, Bulgaria.

Coffee: A total of 60 g of coffee was added to 1 L of demineralized water and boiled for 10 min. Next, the obtained extract of coffee was filtered through a paper filter and supplemented with distilled water to 1 L.

Tea: A total of 10 g of black tea was added to 1 L of demineralized water and boiled for 5 min. Subsequently, the extract of black tea was filtered through a paper filter and supplemented with demineralized water to 1 L.

### 2.3. Discoloration Test

The discoloration of the dental materials was determined by immersing the samples in model liquids and measuring their color changes over time. The samples were stored in the liquids for various time intervals (0.5 h, 1 h, 3 h, 6 h, 24 h, 48 h, 64 h, and 168 h) and then washed and dried to remove any residual liquids. The color of the samples was then measured and compared to that of the reference samples, which were measured prior to immersion tests. The color change over time was used to determine the discoloration of the dental materials.

### 2.4. Characterization of Dental Materials Tested

Color measurement was performed using a KONICA MINOLTA CR-5 spectrophotometer (KONICA MINOLTA, Chiyoda City, Japan) which had an autocalibration function. The dental material samples were measured in reflection mode using a 30 mm port. The results were reported as spectral values, graphs, colorimetric values, color-difference values, pass/fail judgment, pseudo-color, and color assessment. The spectrophotometer measured the spectral range between 360 and 740 nm, using a pulsed xenon lamp as an excitation source.

Optical microscopy was performed using a Keyence VHX-7000 series microscope (Keyence Corporation, Osaka, Japan) in 4K mode. The microscope was equipped with a small high-performance zoom lens VH-Z20R/Z20T, also from Keyence (Keyence Corporation, Osaka, Japan). All images were captured at the same magnification level.

Fourier-transform infrared spectroscopy, FTIR, was used to identify the characteristic functional groups of the initial and discoloration of the dental materials. The IR spectra were obtained using a Vertex 70 spectrometer from Bruker Optics GmbH (Bruker Optics GmbH, Ettlingen, Germany) along with a single-reflection diamond ATR accessory (Platinum ATR) from the same company.

Porous structures were analyzed using a 3FLEX surface characterization analyzer from Micromeritics Instrument Co. (Norcross, GA, USA). The Brunauer–Emmett–Teller (BET) method was used to determine the surface area based on low-temperature N_2_ sorption. The surface area was calculated using the multipoint BET method, analyzing the adsorption data in the relative pressure range of 0.05–0.30 in the *p*/*p*_0_ range.

To evaluate the light-absorption properties, the diffuse reflectance spectra (DRS) were recorded, and the data were converted to obtain the absorption spectra. The measurements were carried out using a Thermo Scientific Evolution 220 (Thermo Fisher Scientific, Waltham, MA, USA) spectrophotometer equipped with a PIN-757 integrating sphere.

The photoluminescence measurements were conducted using a Horiba spectrofluorometer (Horiba, Kyoto, Japan) with a 450 W high-pressure xenon arc lamp as the excitation source. Measurements were carried out at room temperature with a spectral resolution of 2 nm and a slit width of 2 mm, and both the photoluminescence excitation (at λ = 280 nm) and emission spectra were obtained.

## 3. Results

### 3.1. Discoloration Test 

The color parameters of the dental samples were determined using a colorimeter after exposure to liquids such as coffee, tea, and wine. The colorimeter measures the lightness values (L*), a*, and b* that indicate how light or dark a color is, and its location on the green–red and blue–yellow axes in a three-dimensional Cartesian coordinate system. These parameters were then used to calculate the results, which were reported as diffuse reflectance spectra, chroma, *C*, angle of hue, *h*, and the color difference, ΔE, see Table 1. The formulas used to calculate these color parameters are as follows.

**Figure 1:**
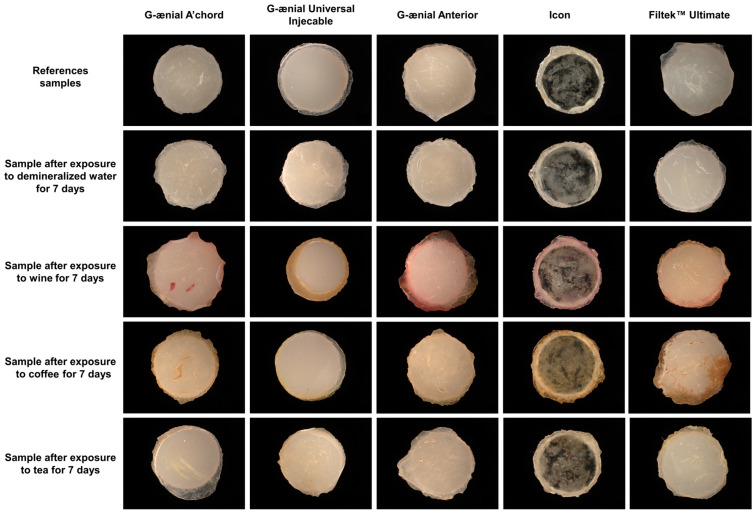
Optical microscopy images of dental samples exposed to demineralized water, wine, coffee, and tea after 7 days.

In the experiment, colorimetric measurements were taken across the entire area of the tested dental material. This was achieved by using optical microscopy to obtain images of the samples. The images were then collected and presented in Figure 1. This approach allowed the average color of the entire area of the material to be determined and quantified.

**Figure 2:**
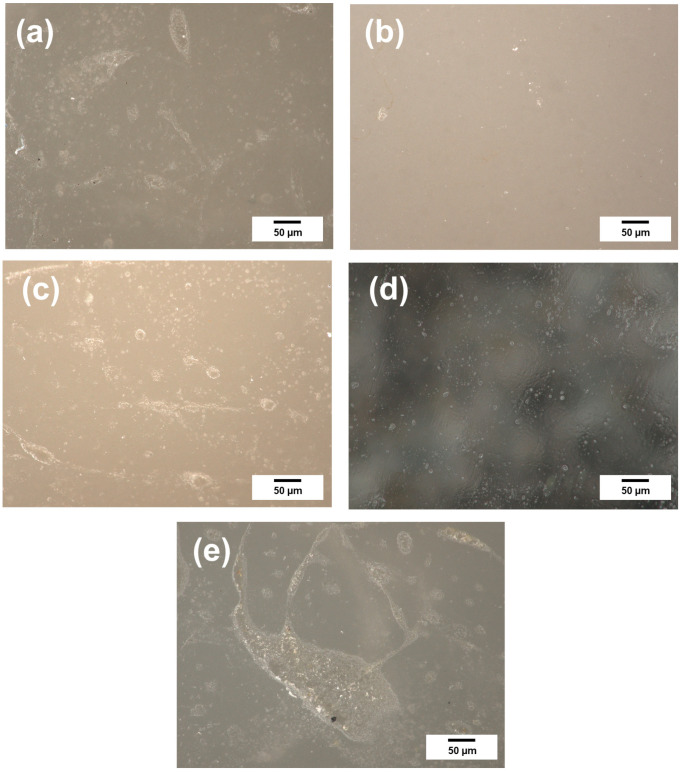
Images of the surface of dental materials: (**a**) G-ænial A’chord (**b**) G-ænial Universal Injectable (**c**) G-ænial Anterior (**d**) Icon, and (**e**) Filtek™ Ultimate.

Optical microscopy was used to analyze surface defects, and 10 samples of each molded composite were analyzed to eliminate manufacturing errors. The results of the analysis are depicted in Figure 2.

### 3.2. Characterization of Functional Groups

**Figure 3:**
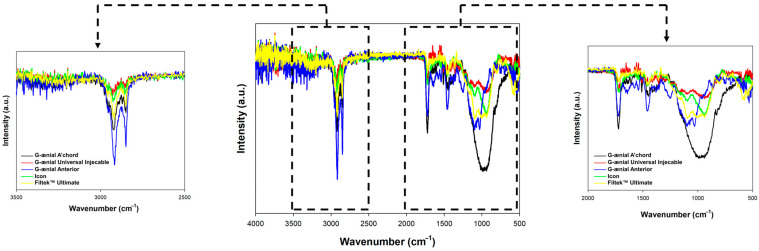
FTIR spectra for analyzed dental materials analyzed, control samples.

To determine the functional groups on the surface of the dental materials, the FTIR-ATR analysis was performed. FTIR-ATR spectra of control samples before any further treatment were collected and are presented in Figure 3.

### 3.3. Textural Properties

The next step of the study considered the critical impact of hydroxyl groups on the surface adsorption process. The evaluation was carried out using a low temperature N_2_ sorption to determine the basic parameters of the porous structure, as shown in Table 2. The evaluation was carried out using low-temperature nitrogen adsorption to determine the basic parameters of the porous structure, including the total pore volume (Vp) and the average pore size (Sp). Vp is the volume of all the pores within a given mass of the material, expressed in cubic centimeters per gram (cm^3^/g), and S p is the average pore size.

### 3.4. Optical Properties 

The results of the optical characterization of the dental materials show that there is a difference in the optical properties between the initial and discolorized materials, seen on both the diffuse reflectance spectra and the luminescence spectra of respected materials.

## 4. Discussion

Colorimetric analysis suggests that coffee and wine had a significant impact on the color saturation of dental samples, leading to a noticeable change in appearance compared to untreated samples. The exact values of the color changes, as well as the correlation with other measurement techniques, can be found in Table 1 and the other data presented in the study.

Immersion of the tested dental materials in coffee or tea does not significantly affect the color angle value or the color saturation value, in contrast to the dental samples colored with wine. According to the available knowledge, the discoloration of dental materials becomes well perceptible for the total color difference, ΔE exceeding 1.0, which was also noted in our earlier work [7]. However, ΔE = 3.3 is the limit value for visual color perception. Discoloration above this value is unacceptable for the use of dental materials. Based on the results of the experiment, the immersion of certain dental materials in coffee and wine caused noticeable discoloration after 7 days of exposure. In particular, the materials G-ænial Anterior, Icon, and Filtek™ Ultimate showed discoloration when exposed to coffee and wine. However, the materials G-ænial A’chord and G-ænial Universal Injectable exceeded the acceptable discoloration limits only when exposed to wine. On the other hand, none of the materials exceeded acceptable discoloration limits when exposed to tea or demineralized water, even after 7 days of exposure. These results suggest that the type of liquid and the specific dental material used can have a notable impact on discoloration over time.

The dental materials tested showed discoloration as a result of coffee and wine. Staining was more significant in areas with micro-cracks, scratches, and rough edges. The discoloration process is classified as adsorption [14]. Therefore, it should be noted that exposed rough edges account for a significant portion of the development of the surface area of dental materials, as also noted by Heintze et al. [15]. In addition, the appearance of cracks, damage, and microbubbles on the coating surface also results in better access to the porous structure of the dental material for the contaminants used (e.g., coffee, wine, etc.) [6].

Optical microscopy was utilized to qualitatively assess surface characteristics, particularly the presence of voids, cracks, and surface irregularities. The G-ænial Universal Injectable and Icon materials exhibited surfaces with minimal cracks, while the other materials (G-ænial A’chord, G-ænial Anterior, and Filtek™ Ultimate) demonstrated more prominent surface defects, such as scratches and micro-cracks. These defects likely contributed to the observed discoloration, rather than serving as a precise measurement of surface roughness. The surface of the G-ænial Universal Injectable and Icon resulted in acceptable levels of discoloration with a maximum of ∆E = 3.3.

FTIR-ATR analysis was carried out to determine the functional groups on the surface of dental materials [16]. The results of the analysis showed that the G-ænial Universal Injectable and Icon materials had a lower concentration of hydroxyl groups compared to the other samples analyzed. The concentration of hydroxyl groups was determined by the intensity of the bands at 3400 cm^−1^ and 1650 cm^−1^ in the obtained spectra [17]. The G-ænial Universal Injectable and Icon materials also showed a lower intensity of the band’s characteristic of the stretching (2900 cm^−1^) and bending (1450 cm^−1^) vibrations of the C-H groups. These results indicate that the dental materials have different concentrations of functional groups on their surfaces, with G-ænial Universal Injectable and Icon materials having a lower concentration of hydroxyl and C-H groups [18]. This information can help to understand the behavior of the materials and their potential for the adsorption of contaminants.

The obtained results of low-temperature nitrogen sorption confirm the earlier hypothesis. It was observed that materials with a smooth surface exhibited a lower specific surface area (approximately 3–4 m^2^/g), and exposure to coffee did not notably affect the tested parameters. The BET surface area and total pore volume remained close to the initial values. Conversely, materials with confirmed surface defects, such as G-ænial Anterior and Filtek™ Ultimate, demonstrated a higher BET surface area (around 10–11 m^2^/g). However, after exposure to coffee, the surface area decreased to 4 m^2^/g. This finding supports the notion that micro-cracks on the surface contribute to the increase in the BET surface area. These micro-cracks are known to be active centers for adsorbing the tested pollutants and are the primary cause of the deterioration of the visual parameters of the dental materials [19].

**Figure 4:**
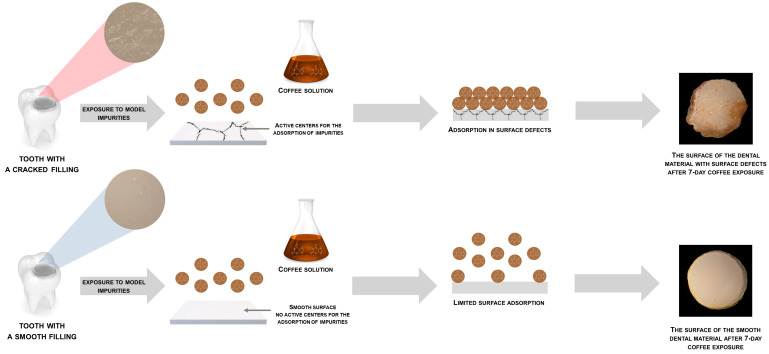
The proposed mechanism for the formation of discoloration on dental materials.

Moreover, the sorption of pollutants like coffee, tea, and wine aligns with the BET multilayer model, as evidenced by the experimental data. Figure 4 presents the proposed mechanism, illustrating this phenomenon. Nevertheless, it is important to note that, to the best of our knowledge, the detailed analysis of the discoloration mechanism of dental materials has not been conducted thus far. Understanding this mechanism is crucial in order to effectively counteract discoloration and prevent its occurrence.

The results of low-temperature nitrogen sorption support the initial hypothesis. Materials with a smooth surface showed a lower specific surface (approximately 3–4 m^2^/g) and were not significantly affected by coffee exposure. The BET surface area and total pore volume remained close to the initial values. However, materials with confirmed surface defects (such as G-ænial Anterior and Filtek™ Ultimate) had a higher BET surface area (approximately 10–11 m^2^/g) and showed a decrease in surface area to 4 m^2^/g after coffee exposure. The results of the study indicate that the presence of micro-cracks on the surface of the dental materials leads to an increase in the BET surface area, making the surface more active for the adsorption of pollutants such as coffee, tea, and wine. The BET multilayer model fits the experimental data for the sorption of these pollutants. The deterioration of the visual parameters of the dental materials, such as discoloration, is mainly due to the presence of these micro-cracks [19]. However, the exact mechanism of discoloration has not yet been analyzed in detail, and more research is needed to fully understand the process and develop strategies to counteract it. This mainly confirms that the micro-cracks on the surface are responsible for the increase in the BET surface area. These sites have been shown to be active centers for the adsorption of the tested pollutants. Additionally, they are the main cause of the deterioration of visual parameters of the dental materials.

The DRS analysis indicates that the absorption spectra of the initial materials are different from those of the discolorized materials, which indicates that the discoloration process influences the optical properties of the materials. The results of the luminescence analysis also indicate that the discoloration process has an effect on the fluorescence of the dental materials, which is confirmed by the change in the fluorescence spectra. Overall, the optical characterization confirms that the discoloration process changes the optical properties of the dental materials.

**Figure 5:**
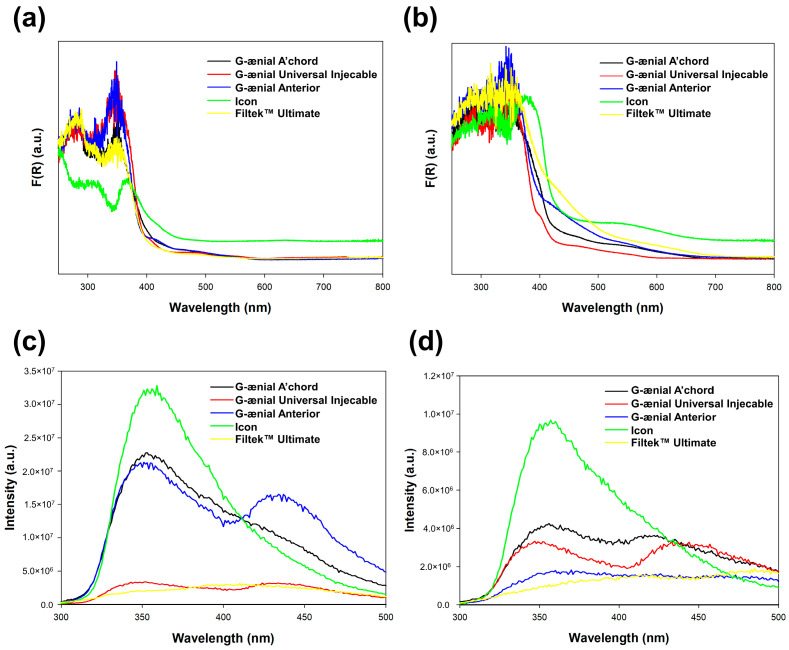
DRS (**a**,**b**) and luminescence (**c**,**d**) spectra for initial dental materials (**a**,**c**) and after 7 days of exposure to a red wine solution (**b**,**d**).

Taking into account the similar shade of the reference dental materials, the DRS spectra are predictably very similar (Figure 5a) [20]. The spectra of the initial materials exhibit a broad spectral absorbance band, with the intensity increasing toward the shorter wavelengths. Only in the case of the Icon material is a slight shift toward longer wavelengths observed. For dental materials immersed in a model solution for 7 days (data are presented for a red wine solution due to a notable color change—Figure 5b), the strongest increase in F(R) was observed for Filtek™ Ultimate and G-ænial Anterior, which is consistent with the data on the discoloration of the samples previously presented. The smallest changes were recorded for the Icon and G-ænial Universal Injectable, which results from the lowest adsorption of the tested model liquids.

The dental materials differed significantly in the intensity of luminescence emitted, which was the highest for Icon and the lowest for Filtek™ Ultimate (Figure 5c) [8]. If the dental sample is treated with the red wine solution (immersed for 7 days), the luminescence intensity was significantly reduced. The strongest luminescence reduction was observed for G-ænial A’chord and G-ænial Anterior. These materials were characterized by high initial luminescence; hence, the observed decrease is due to the high adsorption of the red wine solution on the surface of the tested material. The G-ænial Universal Injectable material was infused with a continuous luminescence, which confirms that its smooth surface prevents the adhesion of impurities to its surface. The different efficiency of the staining agents compared to the tested dental materials is associated with differences in the physical and chemical properties of these materials, in particular the development of the surface area and the number of surface defects. 

Our findings align with previous research indicating that red wine, coffee, and tea can significantly impact the color stability of dental materials. Similar to studies by Villalta et al. and Ertaş et al., our results showed that red wine caused the most pronounced discoloration, followed by coffee and tea [12,21]. The study by Paolone et al. also supports our observation that different liquids have varying staining potentials, with coffee and tea causing more discoloration than demineralized water [4].

The materials G-ænial Universal Injectable and Icon exhibited the least discoloration across all test liquids, consistent with findings by Guler et al. and Mazur-Koczorowska et al., who reported that materials with smoother surfaces and fewer surface defects tend to have better color stability [6,8]. The lower concentration of hydroxyl and C-H groups in these materials, as revealed by FTIR-ATR analysis, may contribute to their reduced propensity for staining, corroborating findings by Hind et al. on the role of surface chemistry in discoloration resistance [16].

The multilayer BET model used in this study to describe the adsorption behavior of discolorants is well established in the literature. The results align with the theoretical framework proposed by Loebenstein and further explored by Sing, which suggests that adsorption on surfaces with higher BET surface areas is more likely to follow a multilayer adsorption pattern [14,22]. This model is particularly relevant for understanding the behavior of materials with surface defects, as these imperfections increase the available surface area for adsorption, thereby enhancing the potential for discoloration [9].

The clinical implications of these findings may be also significant for the selection of dental materials in restorative dentistry; however, more studies are needed to test those material in clinical applications. Materials such as G-ænial Universal Injectable and Icon, which demonstrated superior color stability, may represent the primary interest in such clinical tests, particularly in areas prone to exposure to staining agents like coffee, tea, and red wine.

The observed reduction in surface area after exposure to coffee for materials with initial surface defects underscores the necessity of meticulous finishing and polishing procedures to minimize micro-cracks and surface irregularities. Dental practitioners should be aware that even minor surface imperfections can significantly impact the long-term appearance of restorations [23,24].

Moreover, the findings suggest that the choice of dental materials should consider the specific staining potential of different liquids. For patients who frequently consume staining beverages such as red wine and coffee, selecting materials with demonstrated resistance to these agents could enhance the longevity of aesthetic restorations. This could be particularly relevant in lifestyle-specific recommendations for patients, guiding them towards choices that maintain their dental restorations’ appearance [25].

While the study provides valuable insights, several limitations must be acknowledged. The use of model liquids, although representative, does not entirely replicate the complex environment of the oral cavity, where factors such as salivary enzymes, pH fluctuations, and mechanical wear also influence discoloration. Future studies should incorporate in vivo conditions to better understand the clinical performance of these materials [25].

Another limitation is the relatively short duration of exposure (7 days), which, although sufficient to observe significant discoloration, may not fully capture the long-term effects of staining agents. Longitudinal studies are needed to assess the durability of these materials over extended periods. The study also focused on a limited number of materials and staining agents. Expanding the range of materials and including additional common dietary substances could provide a more comprehensive understanding of the discoloration mechanisms. Additionally, variations in individual patient habits and oral hygiene practices could influence the extent of discoloration, suggesting a need for personalized approaches in material selection [26].

Future research should focus on in vivo studies to validate these findings in clinical settings. Investigating the combined effects of mechanical wear and chemical exposure on discoloration would provide a more comprehensive understanding of material performance. Additionally, exploring the development of new resin formulations with enhanced resistance to staining agents could further improve the aesthetic longevity of dental restorations. Research could also benefit from advanced imaging techniques to better quantify and visualize surface defects and their evolution over time. Understanding the molecular mechanisms underlying the adsorption of different staining agents at the nanoscale could lead to the development of more effective anti-discoloration treatments and coatings for dental materials [27].

Exploring the role of patient-specific factors, such as oral hygiene habits and dietary preferences, in the discoloration process could also provide valuable insights. Personalized dental material recommendations based on individual risk factors for staining could enhance patient satisfaction and outcomes. This study underscores the critical role of surface characteristics in the discoloration of dental materials. Materials with smoother surfaces and fewer defects, such as G-ænial Universal Injectable and Icon, demonstrated superior color stability when exposed to common staining agents. These findings provide a basis for the selection of dental materials in clinical practice to achieve better aesthetic outcomes and highlight the need for ongoing research to develop materials with improved long-term color stability [7].

The insights gained from this study can inform dental practitioners and researchers alike, guiding the development and use of dental materials that meet the high aesthetic demands of patients while maintaining their appearance over time. By considering the impact of surface defects and the adsorption mechanisms of staining agents, future advancements in dental materials can significantly enhance patient satisfaction and the durability of dental restorations. Additionally, the study highlights the need for continuous education for dental professionals on the latest materials and techniques to prevent discoloration. This could involve incorporating findings from current research into dental curricula and ongoing professional development programs. Overall, this study provides a foundation for future innovations in dental materials and clinical practices aimed at achieving and maintaining optimal esthetic results in restorative dentistry.

## 5. Conclusions

Analysis using FTIR-ATR revealed notable variations in the concentrations of functional groups on the surfaces of different dental materials. Specifically, G-ænial Universal Injectable and Icon materials exhibited lower levels of hydroxyl and C-H groups. The presence of distinct functional groups on the surfaces of dental materials can have implications for their behavior and their propensity to adsorb contaminants. Low-temperature N_2_ sorption analysis confirmed that materials with smoother surfaces generally possessed lower specific surface areas. Notably, exposure to coffee led to a reduction in surface area, suggesting that micro-cracks on the material’s surface contribute to the overall increase in the BET surface area. The sorption of common pollutants such as coffee, tea, and wine followed the BET multilayer model, as supported by experimental data. This finding suggests a consistent mechanism for the adsorption of these substances onto dental material surfaces. In summary, the study underscores the significance of considering surface characteristics, including the presence of micro-cracks, when examining the adsorption behavior and potential discoloration of dental materials exposed to contaminants.

## Figures and Tables

**Table 1:** Color parameters of, hue, *h*, chroma, *C*, and the color difference, ΔE, of dental materials exposed to coffee, tea, wine, and distilled water. Pronounced difference, |ΔE| > 6.0; clearly perceptible difference, 3.0 < |ΔE| < 6.0; slightly perceptible difference, 1.7 < |ΔE| < 3.0; imperceptible difference, |ΔE| < 1.7.

Liquid/Time	G-ænial A’chord			G-ænial Universal Injectable			G-ænial Anterior			Icon			Filtek™ Ultimate		
	*C*	*hº*	∆E	*C*	*hº*	∆E	*C*	*hº*	∆E	*C*	*hº*	∆E	*C*	*hº*	∆E
**Water**															
0	29.3	1.42	-	6.03	1.33	-	14.83	1.41	-	1.10	0.43	-	15.55	1.03	-
7 days	18.8	1.43	2.62	2.95	1.25	2.78	10.68	1.45	2.18	1.62	0.77	2.14	8.55	1.32	2.65
**Wine**															
0	23.3	1.47	-	6.39	1.41	-	14.30	1.51	-	1.79	0.80	-	10.46	1.22	-
7 days	19.26	1.32	5.36	9.92	1.52	4.13	22.74	1.50	4.61	1.47	0.78	3.66	21.90	1.47	4.97
**Coffee**															
0	24.05	1.43	-	6.96	1.43	-	18.99	1.53	-	1.69	1.12	-	19.61	1.38	-
7 days	37.17	1.51	3.19	4.91	1.42	3.09	34.71	1.54	4.35	3.72	1.33	3.96	46.16	1.49	4.32
**Tea**															
0	28.57	1.44	-	6.58	1.39	-	16.62	1.45	-	1.43	0.53	-	17.39	1.11	-
7 days	44.4	1.48	3.11	10.64	1.43	3.13	28.06	1.51	3.09	4.20	1.18	3.01	15.79	1.23	3.27

**Table 2:** The parameters of the porous structure for the initial material and after 7 days exposition for coffee.

Sample	A_BET_ (m^2^/g)	V_p_ (cm^3^/g)	S_p_ (nm)
G-ænial Universal Injectable	4	0.01	3.3
G-ænial Universal Injectable (coffee)	3	0.01	3.1
Icon	5	0.02	3.5
Icon (coffee)	4	0.02	3.4
G-ænial Anterior	10	0.04	3.5
G-ænial Anterior (coffee)	2	0.01	3.2
Filtek™ Ultimate	11	0.05	3.7
Filtek™ Ultimate (coffee)	4	0.02	3.3

## Data Availability

The data that support the findings of this study are available from the authors upon reasonable request.
